# Carbetocin for the Prevention of Postpartum Hemorrhage: A Comparative Study

**DOI:** 10.7759/cureus.105672

**Published:** 2026-03-22

**Authors:** Punyashree Manchadas, Manisha M Deshmukh, Mahendra G

**Affiliations:** 1 Anaesthesia, Sri Chamundeshwari Medical College, Hospital & Research Institute, Channapatna, IND; 2 Obstetrics and Gynaecology, Adichunchanagiri Institute of Medical Sciences, Adichunchanagiri University, BG Nagara, IND

**Keywords:** carbetocin, maternal outcomes, oxytocin, postpartum hemorrhage, uterotonics

## Abstract

Introduction: Postpartum hemorrhage (PPH) remains a major obstetric emergency and a leading cause of maternal morbidity and mortality. Uterotonics administered during the third stage of labor play a crucial role in its prevention. Carbetocin has emerged as a potential alternative to oxytocin due to its prolonged uterotonic effect and improved pharmacological profile. The study aimed to evaluate the effectiveness and safety of carbetocin in the prevention of PPH and to compare its outcomes with oxytocin using a combined intramuscular and intravenous oxytocin regimen in women undergoing vaginal or caesarean delivery.

Materials and methods: This prospective observational comparative study was conducted among 100 women (50 in each group) receiving either carbetocin (100 µg intramuscularly) or oxytocin (10 IU intramuscularly with additional intravenous infusion as per institutional protocol) immediately after delivery. Estimated blood loss, incidence of PPH (defined as ≥500 mL for vaginal delivery and ≥1000 mL for cesarean section), fall in hemoglobin, hemodynamic changes, and requirement for additional uterotonics were noted. Statistical analysis was performed, and a p-value <0.05 was considered significant.

Results: Baseline characteristics were comparable between groups. Mean estimated blood loss was significantly lower in the carbetocin group (410 ± 85 mL) compared to the oxytocin group (520 ± 110 mL; p<0.001). PPH occurred in three (6%) women in the carbetocin group versus 12 (24%) women in the oxytocin group (p=0.01). The mean fall in hemoglobin was lower with carbetocin (0.6 ± 0.4 g/dL vs 1.1 ± 0.6 g/dL; p<0.001). Additional uterotonics were required in two (4%) women receiving carbetocin compared to 11 (22%) women receiving oxytocin (p=0.007). Carbetocin was associated with better hemodynamic stability.

Conclusion: Carbetocin was associated with reduced blood loss, lower PPH incidence, and improved hemodynamic stability compared to oxytocin within the context of differing prophylactic regimens. However, larger randomized studies are required to confirm these findings.

## Introduction

Postpartum hemorrhage (PPH) remains one of the most serious and life-threatening obstetric emergencies worldwide and continues to be a leading cause of maternal morbidity and mortality, particularly in low- and middle-income countries [1]. It is defined as blood loss of ≥500 mL following vaginal delivery or ≥1000 mL after caesarean section within the first 24 hours postpartum [2]. Despite advances in obstetric care, PPH accounts for nearly 25-52% of global maternal deaths [3]. The persistent burden of PPH underscores the urgent need for effective preventive strategies that are practical, safe, and applicable across diverse healthcare settings [4].

The etiology of PPH is classically described by the “four Ts”: tone (uterine atony), tissue (retained placental tissue), trauma (genital tract injury), and thrombin (coagulation disorders) [5]. Among these, uterine atony is responsible for approximately 70-80% of cases and represents the most preventable cause [6]. Failure of the uterus to contract effectively after delivery results in continued bleeding from the placental bed, leading to rapid and potentially catastrophic blood loss [7]. Consequently, interventions aimed at ensuring adequate uterine contraction immediately after delivery form the cornerstone of PPH prevention strategies worldwide [8].

Active management of the third stage of labor (AMTSL) has been widely adopted to reduce the incidence of PPH, with prophylactic administration of uterotonic agents being its central component [9]. Oxytocin, administered intramuscularly or intravenously at a dose of 10 IU, is currently recommended by the World Health Organization (WHO) as the first-line uterotonic for prevention of PPH due to its proven efficacy and safety profile [10]. However, oxytocin has certain limitations, including a short half-life of approximately 3-5 minutes, necessitating repeated dosing or continuous infusion to maintain uterine tone [11]. Furthermore, its heat sensitivity requires strict cold-chain storage, which may not be consistently feasible in resource-limited settings [12]. These pharmacokinetic and logistical challenges have prompted exploration of alternative long-acting uterotonic agents [11,12].

Carbetocin, a long-acting synthetic analogue of oxytocin, has emerged as a promising alternative for PPH prophylaxis [13]. Structurally modified to resist enzymatic degradation, carbetocin has a prolonged half-life of approximately 40 minutes, allowing sustained uterine contraction following a single dose [14]. Several clinical studies and systematic reviews have demonstrated that carbetocin is comparable or superior to oxytocin in reducing postpartum blood loss and the need for additional uterotonics after both vaginal and cesarean deliveries [15,16]. Additionally, its favorable safety profile and availability in heat-stable formulations make it particularly attractive for use in varied clinical environments.

However, variations in oxytocin administration protocols, including the use of combined intramuscular and intravenous regimens in routine clinical practice, may influence comparative effectiveness outcomes and should be considered when interpreting results across studies.

In view of these considerations, the present study aimed to evaluate the effectiveness and safety of carbetocin in the prevention of PPH and to compare its outcomes with oxytocin administered as a combined intramuscular and intravenous regimen in women undergoing vaginal or cesarean delivery.

## Materials and methods

This prospective observational comparative study was conducted in the Department of Obstetrics and Gynaecology at Adichunchanagiri Institute of Medical Sciences, Adichunchanagiri University, B.G. Nagara from June 2025 to December 2025 after obtaining approval from the Institutional Ethics Committee [AIMS/IEC/290/2025]. Written informed consent was obtained from all participants prior to enrolment. As this was an observational study, no randomization or blinding was performed, and the choice of uterotonic agent was based on the institutional protocol and the treating obstetrician’s discretion. No fixed standardized allocation criteria were used, and the selection of the uterotonic agent was influenced by clinician judgment, drug availability, and perceived patient risk.

All pregnant women admitted to the labor ward or operation theatre for vaginal delivery or lower segment cesarean section during the study period were screened for eligibility. Women aged 18 years or older, undergoing vaginal or cesarean delivery, with singleton or multiple pregnancies, at term or preterm gestation, and who were hemodynamically stable prior to drug administration were included in the study. Women with known hypersensitivity to carbetocin or oxytocin analogues, those with active bleeding not attributable to uterine atony, and those unwilling to provide consent were excluded.

The sample size was calculated based on previously published data by Priya et al., who reported a mean systolic blood pressure (SBP) at nine minutes of 108.8 ± 8.53 mmHg in the oxytocin group and 116.6 ± 9.74 mmHg in the carbetocin group [17]. Considering a mean difference of 7.8 mmHg and a pooled standard deviation of 9.15 mmHg, the required sample size for comparison of two independent means was calculated using the formula, 

$n = 2 \times \frac{(Z_{\alpha/2} + Z_{\beta})^{2} \sigma^{2}}{\Delta^{2}}$

Assuming a two-sided alpha error of 0.05 and 95% power, the minimum sample size required was 36 participants per group. After adjusting for 15% possible attrition, the sample size increased to 43 per group. For improved statistical robustness and generalizability, the final sample size was rounded to 50 participants in each group, resulting in a total of 100 participants. It is acknowledged that the sample size was calculated based on a secondary hemodynamic parameter rather than the primary clinical outcome, which may limit the statistical power for primary outcome assessment.

Participants were categorized into two groups based on the uterotonic agent received immediately after delivery of the fetus. The oxytocin group received 10 IU oxytocin intramuscularly along with 10 IU diluted in intravenous fluids administered as a slow infusion as per institutional protocol. The intravenous infusion was administered according to routine institutional practice; however, the exact rate and duration were not standardized across all cases. The carbetocin group received a single intramuscular dose of 100 µg carbetocin immediately after delivery. This represents a non-equivalent dosing strategy between groups (single-dose versus combined-route regimen). All women were managed according to standard active management of the third stage of labor [18].

Baseline demographic and obstetric data, including age, parity, gestational age, body mass index, and mode of delivery, were recorded. Hemodynamic parameters, specifically systolic and diastolic blood pressure, were measured using a calibrated automated sphygmomanometer at baseline (immediately before drug administration), and at five minutes and 15 minutes after administration. Estimated blood loss was assessed using calibrated collection drapes and measurement of blood in suction containers after excluding amniotic fluid. Although standardized methods were used, inter-observer variability in blood loss estimation was not formally assessed. The requirement for additional uterotonics, fall in hemoglobin levels (measured pre-delivery and 24 hours post-delivery), and occurrence of PPH (defined as ≥500 mL after vaginal delivery and ≥1000 mL after cesarean section) were documented [19]. Adverse drug reactions, if any, were also recorded.

The primary outcome measure was prevention of PPH as assessed by estimated blood loss and need for additional uterotonics. Secondary outcomes included hemodynamic changes and a fall in hemoglobin levels. No stratified analysis by mode of delivery or other risk factors was performed due to sample size limitations.

Data were entered into Microsoft Excel and analyzed using IBM SPSS Statistics for Windows, Version 26 (Released 2018; IBM Corp., Armonk, New York, United States). Continuous variables were expressed as mean ± standard deviation and compared using an independent sample t-test or repeated measures ANOVA, where applicable. Categorical variables were expressed as frequency and percentage and compared using the chi-square test. A p-value of less than 0.05 was considered statistically significant. No multivariable adjustment or propensity score analysis was performed to control for potential confounding.

## Results

The baseline characteristics were comparable between the two groups. The majority of participants belonged to the 21-25 years age group, accounting for 28 (56%) in the carbetocin group and 27 (54%) in the oxytocin group. The mean age was 24.8 ± 3.5 years in the carbetocin group and 24.6 ± 3.2 years in the oxytocin group (p=0.77). Mean BMI was similar in both groups (24.1 ± 2.8 vs 24.4 ± 3.1 kg/m²; p=0.61). Primigravida women constituted 30 (60%) in the carbetocin group and 29 (58%) in the oxytocin group (p=0.84), indicating no statistically significant baseline difference between groups (Table 1).

**Table 1:** Baseline Demographic Characteristics This table presents the baseline demographic variables of participants in the carbetocin and oxytocin groups, including age distribution, body mass index (BMI), and parity status. Categorical variables are expressed as number and percentage (N (%)), while continuous variables are presented as mean ± standard deviation (SD). Comparison between groups was performed using the chi-square (χ²) test for categorical variables and the independent (unpaired) t-test for continuous variables. A p-value of less than 0.05 was considered statistically significant. BMI: Body Mass Index; SD: Standard Deviation; χ²: Chi-square Test.

Variable		Carbetocin (n=50)	Oxytocin (n=50)	Total (n=100)	Statistical Value	p-value
Age (years)	≤20	4 (8.0%)	5 (10.0%)	9 (9.0%)	χ² = 0.36	0.95
	21–25	28 (56.0%)	27 (54.0%)	55 (55.0%)		
	26–30	14 (28.0%)	15 (30.0%)	29 (29.0%)		
	31–35	4 (8.0%)	3 (6.0%)	7 (7.0%)		
	Mean ± SD	24.8 ± 3.5	24.6 ± 3.2	24.7 ± 3.3	t = 0.29	0.77
BMI (kg/m²)	Mean ± SD	24.1 ± 2.8	24.4 ± 3.1	24.3 ± 2.9	t = 0.51	0.61
Primigravida	N (%)	30 (60%)	29 (58%)	59 (59%)	χ² = 0.04	0.84

Normal vaginal delivery was the predominant mode of delivery in both groups, observed in 33 (66%) women in the carbetocin group and 32 (64%) women in the oxytocin group, while LSCS was performed in 17 (34%) and 18 (36%) women, respectively. There was no statistically significant difference in mode of delivery between the two groups (p=0.84) (Table 2).

**Table 2:** Mode of Delivery This table shows the distribution of participants according to the mode of delivery in the carbetocin and oxytocin groups. Data are presented as number and percentage (N (%)) of women undergoing normal vaginal delivery (NVD) or lower segment cesarean section (LSCS). The comparison between the two groups was carried out using the chi-square (χ²) test. NVD: Normal Vaginal Delivery; LSCS: Lower Segment Cesarean Section; χ²: Chi-square Test. A p-value of less than 0.05 was considered statistically significant.

Mode of Delivery	Carbetocin (n=50)	Oxytocin (n=50)	Total (n=100)	χ² value	p-value
NVD	33 (66%)	32 (64%)	65 (65%)	0.04	0.84
LSCS	17 (34%)	18 (36%)	35 (35%)		

In the carbetocin group, there was no statistically significant change in SBP or DBP across 0, five, and 15 minutes (p=0.41 and p=0.33, respectively), demonstrating hemodynamic stability. In contrast, the oxytocin group showed a statistically significant reduction in both SBP and DBP over time (p=0.01 and p=0.008 respectively), indicating a notable intragroup fall in blood pressure following administration (Table 3).

**Table 3:** Intragroup Comparison of Blood Pressure This table presents the intragroup comparison of systolic blood pressure (SBP) and diastolic blood pressure (DBP) at different time intervals (0, five, and 15 minutes) following drug administration within each study group. Values are expressed as mean ± standard deviation (SD). Repeated measures analysis of variance (ANOVA) was applied to evaluate changes over time within each group, and the F statistic with corresponding p-value is reported. SBP: Systolic Blood Pressure; DBP: Diastolic Blood Pressure; SD: Standard Deviation; ANOVA: Analysis of Variance. A p-value of less than 0.05 was considered statistically significant.

Group	Parameter	0 min (Mean ± SD)	5 min	15 min	Statistical Value (F)	P-value
Carbetocin	SBP	118.4 ± 9.8	117.2 ± 9.2	116.8 ± 8.7	F = 0.89	0.41
	DBP	78.2 ± 6.4	75.6 ± 7.1	76.8 ± 6.3	F = 1.12	0.33
Oxytocin	SBP	119.2 ± 11.9	113.6 ± 12.4	112.8 ± 10.5	F = 4.82	0.01
	DBP	78.0 ± 7.8	72.8 ± 8.9	73.2 ± 8.1	F = 5.14	0.008

On intergroup comparison, no significant difference in SBP or DBP was observed at 0 minutes and five minutes. However, at 15 minutes, SBP (116.8 ± 8.7 vs 112.8 ± 10.5 mmHg; p=0.038) and DBP (76.8 ± 6.3 vs 73.2 ± 8.1 mmHg; p=0.021) were significantly lower in the oxytocin group compared to the carbetocin group, suggesting relatively better hemodynamic stability in the carbetocin group (Table 4).

**Table 4:** Intergroup Comparison of Blood Pressure This table illustrates the comparison of systolic and diastolic blood pressure between the carbetocin and oxytocin groups at different time intervals (0, five, and 15 minutes). Values are presented as mean ± standard deviation (SD). Intergroup comparisons were performed using the independent (unpaired) t-test, and the corresponding t-values and p-values are shown. SBP: Systolic Blood Pressure; DBP: Diastolic Blood Pressure; SD: Standard Deviation. A p-value of less than 0.05 was considered statistically significant.

Time	Parameter	Carbetocin (Mean ± SD)	Oxytocin (Mean ± SD)	Statistical Value (t)	P-value
0 min	SBP	118.4 ± 9.8	119.2 ± 11.9	0.36	0.71
	DBP	78.2 ± 6.4	78.0 ± 7.8	0.14	0.88
5 min	SBP	117.2 ± 9.2	113.6 ± 12.4	1.66	0.09
	DBP	75.6 ± 7.1	72.8 ± 8.9	1.76	0.08
15 min	SBP	116.8 ± 8.7	112.8 ± 10.5	2.10	0.038
	DBP	76.8 ± 6.3	73.2 ± 8.1	2.34	0.021

Both groups demonstrated a significant decline in hemoglobin levels post-delivery (p<0.001 in both groups). However, the mean fall in hemoglobin was significantly lower in the carbetocin group (0.6 ± 0.4 g/dL) compared to the oxytocin group (1.1 ± 0.6 g/dL), with a statistically significant intergroup difference in post-delivery hemoglobin levels (p=0.004) (Table 5).

**Table 5:** Hemoglobin Levels This table presents the pre-delivery and post-delivery hemoglobin levels in both groups along with the mean fall in hemoglobin concentration. Values are expressed as mean ± standard deviation (SD). Within-group comparison of pre- and post-delivery hemoglobin was performed using the paired t-test, while intergroup comparison was carried out using the independent (unpaired) t-test. Hb: Hemoglobin; SD: Standard Deviation. A p-value of less than 0.05 was considered statistically significant.

Group	Pre-delivery Hb (Mean ± SD)	Post-delivery Hb	Mean Fall	Statistical Value (Paired t)	P-value
Carbetocin	12.4 ± 1.1	11.8 ± 0.9	0.6 ± 0.4	t = 6.92	<0.001
Oxytocin	12.2 ± 1.2	11.1 ± 1.3	1.1 ± 0.6	t = 9.14	<0.001
Statistical Value (unpaired t)	t = 0.88	2.91			
p-value	t = 0.38	0.004			

The requirement of additional uterotonics was significantly lower in the carbetocin group, required in only two (4%) women compared to 11 (22%) women in the oxytocin group (p=0.007), indicating a lower requirement for additional uterotonics in the carbetocin group (Table 6).

**Table 6:** Requirement of Additional Uterotonics This table depicts the frequency of the requirement of additional uterotonic agents in the carbetocin and oxytocin groups. Data are presented as number and percentage [N (%)]. The difference between the groups was assessed using the chi-square (χ²) test. χ²: Chi-square test. A p-value of less than 0.05 was considered statistically significant.

Additional Uterotonics	Carbetocin (n=50)	Oxytocin (n=50)	χ² value	p-value
Yes	2 (4%)	11 (22%)	7.21	0.007
No	48 (96%)	39 (78%)		

Mean estimated blood loss was significantly lower in the carbetocin group (410 ± 85 mL) compared to the oxytocin group (520 ± 110 mL) (p<0.001). Furthermore, PPH (defined as ≥500 mL for vaginal delivery and ≥1000 mL for cesarean section) occurred in three (6%) women in the carbetocin group versus 12 (24%) women in the oxytocin group (p=0.01), demonstrating a clinically and statistically significant reduction in blood loss with carbetocin (Table 7).

**Table 7:** Estimated Blood Loss This table shows the comparison of mean estimated blood loss and the occurrence of postpartum hemorrhage (PPH) between the carbetocin and oxytocin groups. Continuous variables are presented as mean ± standard deviation (SD), while categorical variables are expressed as number and percentage (N (%)). Intergroup comparison of mean blood loss was performed using the independent (unpaired) t-test, and the difference in incidence of PPH was analyzed using the chi-square (χ²) test. A p-value of less than 0.05 was considered statistically significant. PPH: Postpartum Hemorrhage; SD: Standard Deviation; χ²: chi-square test.

Parameter	Carbetocin	Oxytocin	Statistical Value	p-value
Mean Blood Loss (mL)	410 ± 85	520 ± 110	t = 5.59	<0.001
PPH (>500 mL)	3 (6%)	12 (24%)	χ² = 6.45	0.01

No significant adverse drug reactions were observed in either group during the study period.

## Discussion

The present study demonstrated that carbetocin was associated with a significantly lower incidence of PPH compared to oxytocin, with PPH (defined as ≥500 mL for vaginal delivery and ≥1000 mL for cesarean section) occurring in 3(6%) women in the carbetocin group versus 12(24%) in the oxytocin group (p=0.01). These findings are comparable to those reported by Pathak et al., who observed PPH in 4 (5.5%) women receiving carbetocin compared to 16 (22.2%) in the oxytocin group, showing a statistically significant reduction with carbetocin [16]. Similarly, Kang et al. reported a lower frequency of PPH in the carbetocin group, reinforcing the sustained uterotonic efficacy of carbetocin [20]. However, it should be noted that differences in dosing protocols and study populations across studies may influence the comparability of results. The consistency of these findings across studies suggests that carbetocin may provide effective prophylaxis against uterine atony-related bleeding.

In terms of estimated blood loss, the present study showed a significantly lower mean blood loss in the carbetocin group (410 ± 85 mL) compared to the oxytocin group (520 ± 110 mL; p<0.001). PPH was also less frequent with carbetocin. Similar results were reported by Voon et al. and Arif et al., who documented reduced intraoperative and postpartum blood loss in women receiving carbetocin [21,22]. Pathak et al. also noted a higher proportion of women with lower blood loss volumes in the carbetocin group [16]. While these findings are consistent, the observed differences in the present study should be interpreted cautiously in view of the non-equivalent uterotonic regimens used. These comparable observations support the longer duration of uterine contractility achieved with carbetocin, resulting in improved control of postpartum bleeding.

The requirement for additional uterotonics was significantly lower in the carbetocin group in the present study, with only two (4%) women requiring additional agents compared to 11 (22%) in the oxytocin group (p=0.007). This finding parallels the study by Pathak et al., where additional uterotonics were required in 13 (17.8%) women in the carbetocin group compared to 41 (56.9%) in the oxytocin group [16]. Similarly, Tse et al. reported lower additional uterotonic use in the carbetocin group compared to oxytocin [23]. The reduced requirement for rescue therapy observed consistently across studies highlights the sustained uterotonic effect of carbetocin following a single dose, although the influence of differing administration protocols cannot be excluded.

With respect to hemoglobin levels and hemodynamic stability, the present study showed a significantly smaller fall in hemoglobin in the carbetocin group (0.6 ± 0.4 g/dL) compared to the oxytocin group (1.1 ± 0.6 g/dL; p<0.001). Moreover, significant reductions in systolic and diastolic blood pressure were observed in the oxytocin group but not in the carbetocin group. Similar findings were reported by Alam et al., who documented a greater post-delivery hemoglobin decline in women receiving oxytocin compared to carbetocin [24]. Larciprete et al. also observed more pronounced hypotensive effects with oxytocin infusion, whereas carbetocin maintained more stable hemodynamic parameters [25]. These comparable findings suggest that carbetocin may be associated with improved hemodynamic stability, although causal inference is limited by the observational study design.

Strengths and limitations

The present study has several strengths, including an adequate sample size of 100 participants with equal group distribution, prospective data collection, and comprehensive assessment of clinically relevant outcomes such as estimated blood loss, hemoglobin fall, hemodynamic changes, and requirement of additional uterotonics. The inclusion of both vaginal and cesarean deliveries enhances the generalizability of the findings to routine obstetric practice.

However, several important limitations must be acknowledged. First, the study compared a single-dose intramuscular carbetocin regimen with a combined intramuscular and intravenous oxytocin protocol, representing non-equivalent dosing strategies that may influence outcome interpretation. Second, allocation of a uterotonic agent was based on clinician's discretion rather than randomization, introducing potential selection bias and confounding. Third, the study population was heterogeneous, including vaginal and cesarean deliveries, singleton and multiple pregnancies, and term and preterm gestations, without stratified analysis, which may limit clinical interpretability. Fourth, the sample size was calculated based on a secondary hemodynamic parameter rather than the primary clinical outcome. Additionally, blood loss estimation, although performed using standardized methods, may be subject to measurement variability, and the study was conducted at a single tertiary care center, which may limit external validity. Larger multicentric randomized trials would further strengthen the evidence regarding the comparative effectiveness of carbetocin and oxytocin.

## Conclusions

The findings of this prospective observational study suggest that carbetocin may be associated with lower estimated blood loss, reduced incidence of PPH, smaller decline in hemoglobin levels, decreased requirement for additional uterotonics, and better hemodynamic stability when compared to oxytocin in women undergoing vaginal or cesarean delivery. Although these results indicate a potential clinical advantage of carbetocin in the prevention of PPH, the observational design of the study precludes establishing definitive causality. Further large-scale randomized controlled trials are warranted to confirm these findings and to strengthen the evidence for routine use of carbetocin in obstetric practice.
